# Compounded hormonal pellets: a critical review of current evidence and risks

**DOI:** 10.1590/1806-9282.20250121

**Published:** 2025-08-08

**Authors:** Maria Celeste Osório Wender, Marcelo Luis Steiner, César Eduardo Fernandes, Lia Cruz Vaz da Costa Damásio, José Maria Soares, Lúcia Helena Simões da Costa Paiva, Ilza Maria Urbano Monteiro, Ricardo de Almeida Quintairos, Clayton Luiz Dornelles Macedo, Agnaldo Lopes da Silva

**Affiliations:** 1 Universidade Federal do Rio Grande do Sul, Faculty of Medicine, Department of Gynecology and Obstetrics – Porto Alegre (RS), Brazil.; 2 Faculdade de Medicina do ABC, Department of Gynecology – Santo André (SP), Brazil.; 3 Universidade Federal do Piauí, Faculty of Medicine, Department of Gynecology – Teresina (PI), Brazil.; 4 Universidade de São Paulo, Faculty of Medicine, Department of Obstetrics and Gynecology – São Paulo (SP), Brazil.; 5 Universidade Estadual de Campinas, Faculty of Medical Sciences, Department of Obstetrics and Gynecology – Campinas (SP), Brazil.; 6 Center of Endometriosis – Belém (PA), Brazil.; 7 Universidade Federal de São Paulo, Núcleo de Endocrinologia do Exercício – São Paulo (SP), Brazil.; 8 Universidade Federal de Minas Gerais, Faculty of Medicine, Department of Gynecology and Obstetrics – Belo Horizonte (MG), Brazil.

## INTRODUCTION

The use of compounded hormonal pellets has recently gained popularity in Brazil, driven by claims of benefits beyond their intended clinical indications, such as enhanced muscle mass, improved physical and sexual performance, and overall well-being. However, this growing trend raises significant concerns within the medical community due to the lack of robust scientific evidence supporting their safety and efficacy in the treatment of gynecological conditions, as well as insufficient regulatory oversight.

The only hormonal pellet approved by the National Health Surveillance Agency (ANVISA) is Implanon^®^ (Nexplanon^®^), which contains etonogestrel and is used as a contraceptive^1^. This pellet has undergone extensive clinical studies for safety and efficacy. In contrast, other pellets like gestrinone and testosterone are made in compounding pharmacies without strict dosage control or strong evidence of safety, particularly for non-medical uses. Health organizations warn of increased adverse effects linked to the indiscriminate use of these compounded hormonal pellets.

This article aimed to review the available scientific evidence on the safety and efficacy of hormones used in subdermal pellets.

## METHODS

This non-systematic literature review critically evaluates the efficacy, safety, and clinical implications of hormonal pellets. The search was conducted in PubMed and Google Scholar, using terms such as "subdermal pellets," "estradiol," "gestrinone," "testosterone," "nestorone," "oxytocin," and "oxandrolone pellets." Studies in English, with no date restrictions, were included, and the search concluded in November 2024.

Original studies, systematic reviews, meta-analyses, and randomized clinical trials were analyzed, while those with incomplete data or irrelevant to the review were excluded. Titles and abstracts were screened, and selected articles were fully reviewed. References were manually examined for additional relevant studies.

Key data on study population, intervention, dosage, treatment duration, outcomes, results, and adverse effects were extracted. Study quality was assessed based on design, sample size, methodology, and clinical applicability. The GRADE system was applied to evaluate the evidence quality of compounded hormonal pellets (Table 1), grouping studies by hormone type and clinical outcomes, including contraceptive efficacy, symptom relief, and adverse effects^2^. All articles collected and reviewed for this narrative are shown in Table 2.

**Table 1:** Quality of evidence, definitions, and implications.

Grade level	Definition	Implications
High	Evidence is derived from consistent results from well-designed randomized controlled trials (RCTs) or overwhelming observational studies.	Further research is unlikely to change confidence in the estimate of effect.
Moderate	Evidence comes from RCTs with some limitations or strong evidence from well-conducted observational studies.	Further research may have an impact on confidence in the estimate of effect and may change the result.
Low	Evidence is based on observational studies or RCTs with serious limitations (e.g., risk of bias, inconsistency, and indirectness).	Further research is likely to have an important impact on confidence in the estimate of effect and is likely to change the result.
Very low	Evidence is based on case series, expert opinions, or studies with very serious limitations.	Any estimate of the effect is very uncertain.

**Table 2:** Articles collected and reviewed for this narrative.

Authors	Study design	Population	Intervention	Comparison	Outcome	Adverse effects
Coutinho et al.^4^	Prospective observational study	531 reproductive-age women	Pellets with gestrinone (2–5 pellets, 30–40 mg each)	–	Pregnancy within 8 months postinsertion	Amenorrhea in 30%
Diaz et al.^5^	Prospective observational study	38 fertile, normally menstruating women	Six pellets with gestrinone (30 mg per pellet)	–	High contraceptive efficacy	Amenorrhea, acne, nervousness; elevated transaminases
Alvarez et al.^6^	Non-randomized comparative study	100 women using contraceptive pellets	Pellets with gestrinone (30.1 mg per pellet) (n=48)	Levonorgestrel pellets (n=52)	Oligomenorrhea in 80–90%; elevated hematocrit	Elevated transaminases, itching, and acne
Brincat et al.^10^	Randomized controlled trial	55 postmenopausal women	Pellets with estradiol+testosterone	–	Relief of climacteric symptoms	–
Buckler et al.^11^	Randomized controlled trial	30 postmenopausal women	Testosterone pellets, 100 mg (n=15)	Subcutaneous placebo (n=15)	Testosterone peak in 1 month	No reported adverse effects
Glaser et al.^13^	Prospective study using a self-administered Health-Related Quality of Life questionnaire (Menopause Rating Scale [MRS]).	300 pre- and postmenopausal women with symptoms of androgen deficiency	Testosterone pellets, with an average dose of 121 mg	Symptom scores at baseline compared to 3 months post-therapy (no control group)	Significant improvement in somatic, psychological, and urogenital symptoms measured by MRS. Greater response in patients with more severe baseline symptoms	Slight increases in facial hair and mild acne (4.4%), mild increases in aggression or irritability. No serious adverse events. Three patients discontinued therapy due to a perceived lack of efficacy
Glaser et al.^12^	Prospective observational study	27 women with migraines	Testosterone pellets, with an average dose of 130 mg	–	92% improvement in migraines	No related adverse effects
Glaser et al.^14^	Prospective observational study	297 women	Testosterone pellets, with an average dose of 133.3 mg/fixed dose of 100 mg	–	Improvement in androgenic symptoms	11.2% with acne. 1% voice changes. Wide variation in serum levels besides identical dosing
Glaser et al.^15^	Prospective cohort study	1,268 women	Testosterone (T) or T+anastrozole pellets, 55–240 mg	Initial control with T (n=119)	Reduction in breast cancer incidence	Mild/moderate androgenic effects
Glaser et al.^16^	Prospective cohort study	1,267 women	Testosterone (T) or T+anastrozole pellets	SEER (breast cancer data)	Breast cancer incidence lower than the expected SEER rate	Mild androgenic effects
Donovitz^17^	Retrospective cohort study	376,254 patients (85% of women with pre- and postmenopausal age of 25–92 years)	Testosterone and/or estradiol pellets, with adjusted doses	Historical adverse effects	43% of patients discontinued pellet therapy after the initial pellet insertion. 93.3% continuity rate after two pellet insertion	Complications <1%
Stanczyk et al.^18^	Prospective comparative study	20 postmenopausal women with total hysterectomy	Two subdermal pellets with 25 mg estradiol	Transdermal estradiol patch	Consistent estradiol levels with pellets; greater FSH suppression	Breast sensitivity (pellets) and skin reactions (patches)
Suhonen et al.^22^	Prospective cohort study	10 postmenopausal women with climacteric symptoms	Estradiol valerate (E2V) pellets, 3×12 mg	Estradiol benzoate (E2B) pellets, 4×27 mg	Consistent FSH suppression (E2V); symptom return in 8–10 months	Increased breast density; bleeding in the E2B group
Suhonen et al.^21^	Prospective cohort study	36 postmenopausal women with climacteric symptoms	One or three estradiol pellets+IUD with levonorgestrel	–	Stable estradiol concentrations with three pellets	Mild discomfort; positive acceptance of simplicity
Forsling et al.^20^	Observational study	60 women with hysterectomy	Estradiol pellets (50 mg) with/without testosterone (100 mg) or patches	Hysterectomy groups with/without oophorectomy	Increased vasopressin in estradiol groups	Fatigue
Panay et al.^19^	Prospective comparative study	44 women with hysterectomy and bilateral oophorectomy	Subcutaneous estradiol pellets, 25 mg	Subcutaneous estradiol pellets, 50 mg	Higher estradiol levels with 50 mg, no difference in symptoms	Hot flashes and vaginal dryness (50 mg); mild depression
Vashisht and Studd^25^	Prospective cohort study	18 postmenopausal women with low bone density	Estradiol 50 mg every 6 months	Untreated group	Increased bone density after 5 years	–
Filho et al.^24^	Retrospective cohort study	258 postmenopausal women without hysterectomy	Estradiol (4–6×50 mg/year) + testosterone (2×50 mg/year)	–	17% with endometrial thickening; increased polyps and hyperplasia	No endometrial cancer; polyps (38.6%); hyperplasia (20.5%)
Barbosa et al.^23^	Prospective study	40 postmenopausal women	Estradiol pellets (4×17-β estradiol) or estradiol+nomegestrol	–	Reduction of climacteric symptoms and improvement in urogenital symptoms	Mild mastalgia and hyperemic vagina
Britto et al.^26^	Prospective cohort study	61 postmenopausal women	Estradiol (4×50 mg) and testosterone (2×40 mg)	–	Increased BMD in spine and femur (pellet group)	Mild weight gain in both groups
Coutinho et al.^27^	Prospective cohort study	285 reproductive-age women	ST-1435 pellets (3–5 capsules)	–	No pregnancies (except for three cases in Groups B and C); changes in bleeding patterns	Irregular menstrual bleeding
Díaz et al.^28^	Prospective cohort study	70 fertile women, with a mean age of 27 years	Nestorone pellet (76–82 mg)	Copper IUD	No pregnancies; ovulation inhibition	Irregular bleeding; enlarged follicles, slight bilirubin elevation
Coutinho et al.^29^	Prospective cohort study	135 lactating women	Elcometrine 50 mg, replaced every 6 months	Copper IUD	No pregnancies; high breastfeeding rate, low Elcometrine levels in infants	Irregular menstrual bleeding
Brache et al.^30^	Dose-response clinical trial	120 women, aged 18–38 years	One or two Nestorone pellets	–	Complete ovulation inhibition in the first 6 months	Estrogen suppression
Fraser et al.^32^	Prospective clinical study	110 women, aged 18–38 years	Norplant, Nestorone (80–120 mg), or vaginal ring	–	Reduced menstrual volume in all groups	Bleeding changes, increased amenorrhea and oligomenorrhea
Massai et al.^33^	Prospective cohort study	200 lactating women	Nestorone pellets, 80 mg, release of 100 μg/day	Copper T-IUD	No pregnancies; extended amenorrhea and prolonged lactation	Prolonged bleeding, headache, menstrual irregularity
Ylänen et al.^31^	Open-label, non-randomized clinical trial	21 women with endometriosis	Nestorone pellets, 150, 200, or 400 μg/day	–	Significant reduction in pelvic pain	Bleeding/spotting, hot flashes, headache, acne, insomnia

IUD: intrauterine device; SEER: surveillance epidemiology and end results; FSH: follicle-stimulating hormone; BMD: bone mass density; T-IUD: T-shaped intrauterine device.

## RESULTS

### Gestrinone

Gestrinone, or ethylnorgestrienone or progestogen R-2323, is an antigonadotropic agent with antiestrogenic, androgenic, and antiprogesterone effects. It has been explored since the 1980s for the oral treatment of endometriosis. Although it first appeared as a contraceptive, it has not advanced due to factors such as high cost and lack of significant advantages compared to other methods^3^.

Only three trials were identified for gestrinone pellets indicating the lack of good-quality data to assess the safety and efficacy profile of this delivery method. The studies by Coutinho et al.^4^, Diaz et al.^5^, and Alvarez et al.^6^, investigated the contraceptive efficacy and adverse effects of these pellets in women of different ages and reproductive characteristics.

Coutinho et al.^4^ studied 531 women using 2–5 subdermal pellets, noting pregnancy rates varied by capsule count, with most pregnancies occurring after 8 months. About 30% experienced amenorrhea. Diaz et al.^5^ found that in 38 women, pellets effectively inhibited fertility but often caused amenorrhea, androgenic effects (acne, hypertrichosis), headaches, and nervousness. After 3 months, 20 of 27 women had elevated transaminases. Alvarez et al.^6^ compared the efficacy of gestrinone and levonorgestrel pellets in women using six capsules and found that 80–90% of gestrinone users had oligomenorrhea or amenorrhea, plus increased transaminases, pruritus, and acne. Hematocrit levels were higher in gestrinone users, with significant differences at 3 and 12 months (p<0.01). The study ended early due to transaminase concerns from the Diaz study.

Three ongoing clinical trials are investigating gestrinone. One assesses its efficacy in relieving pelvic pain from deep endometriosis (NCT05570786). Another compares gestrinone and dienogest for symptom reduction and disease progression in endometriosis (NCT06543550). The third examines its impact on endothelial function in young patients with cardiovascular disease (NCT06402344).

The evidence on the use of gestrinone pellets is very ­limited and of very low quality, primarily due to methodological ­limitations, small sample sizes, and the age of the available ­studies. While recent clinical trials are ongoing to investigate the potential of bioabsorbable gestrinone pellets, current evidence is insufficient to support their safe and effective use.

### Testosterone

Testosterone plays a vital role in female physiology, particularly influencing sexual desire in postmenopausal women. Its use is currently approved only for specific sexual dysfunction cases, with guidelines recommending physiological doses to prevent hyperandrogenism and side effects. In the US and elsewhere, testosterone is often used off-label when other treatments fail^7^. Clinical trials on testosterone for postmenopausal women show mixed results, with some suggesting benefits for desire and satisfaction, especially in those with lower androgen levels, like postmenopausal women or those who have undergone oophorectomy^8,9^.

The effectiveness of compounded testosterone pellets is inconclusive and hence not recommended due to the risks of high testosterone levels, which can lead to adverse effects^8^. Studies on these pellets are limited by small sample sizes and lack rigorous design, focusing more on outcomes like breast cancer rather than general safety. A 2019 review highlighted the low-quality evidence regarding testosterone's benefits and safety in women, concluding that current data do not support widespread use^8^. Reported side effects include acne, increased oiliness, and hirsutism, with higher doses potentially causing more serious issues like voice changes and mood swings. While specific cardiovascular risks of pellets are not well documented, testosterone is known to increase cardiovascular event risks, particularly at high levels^9^. Overall, the evidence for testosterone pellets in women is limited and of low quality, necessitating a cautious approach to their use.

The evidence supporting testosterone pellets in women is limited and of low quality, with methodological flaws and inconsistent findings. Most studies are observational, with few small randomized trials. Brincat et al. noted climacteric symptom improvements, while Buckler et al. reported enhanced sexual function, but both lacked adverse effect data^10,11^.

A pilot study indicated that testosterone pellets (100–160 mg) might relieve migraines, but it comprised only 27 participants, had no control group, and used a non-validated questionnaire, undermining its reliability. The same authors later noted symptom improvements related to "androgen deficiency" in pre- and postmenopausal women, but the lack of placebo control and structured side-effect assessment compromised validity. Another study showed significant variability in testosterone levels post-therapy and mild androgenic side effects like acne and hair growth. Glaser and Dimitrakakis suggested a lower breast cancer incidence in testosterone users, but methodological flaws, such as poor control groups and non-randomized design, hindered conclusive findings. A 10-year follow-up study on testosterone with or without aromatase inhibitors also had critical issues, including an unmatched control group. Donovitz reported a high dropout rate after initial pellet use, which improved after a second insertion but lacked a thorough side-effect assessment. A major concern with testosterone pellets is their potential to induce supraphysiological hormone levels, increasing risks like voice changes, weight gain, and mood disturbances. Due to the lack of robust evidence, dosing variability, and unresolved safety issues, further rigorous studies are needed to assess their efficacy and safety, especially for esthetic or non-regulated uses.

### Estradiol

Estradiol plays a key role in gynecological and menopausal management. The estradiol pellet Riselle^®^, once available in Brazil and other countries, met regulatory standards before its global discontinuation in 2012.

In general, they help maintain stable estradiol levels, as noted by Stanczyk^18^ and Panay^19^, but can cause breast tenderness, skin reactions, and fatigue. Forsling^20^ found that estradiol increases vasopressin, while testosterone suppresses it, indicating possible effects on water balance and kidney function, relevant for patients with renal or cardiovascular issues.

Suhonen^21,22^ and Barbosa^23^ reported gynecological side effects, including prolonged bleeding, endometrial hyperplasia, and polyps, especially at higher doses. Vashisht^24^ and Britto^25^ suggested a protective effect on bone health, but high doses require caution due to adverse effects.

Evidence on compounded estradiol pellets is limited and of low quality, mainly from observational studies and small trials. While they offer some benefits, the lack of robust trials and safety concerns restricts their clinical use, particularly for off-label or esthetic purposes.

### Nestorone

Nestorone (segesterone acetate or Elcometrine) is a synthetic progestin with high progesterone receptor affinity and no significant androgenic activity. The Annovera^®^ vaginal ring, combining segesterone acetate and ethinyl estradiol, is approved for contraception in the US. However, its off-label use for esthetic purposes raises concerns about safety and prolonged exposure without a clear clinical indication.

Our search identified seven clinical studies on Nestorone pellets. Early research on Elcometrine (ST-14350) reported high rates of bleeding pattern changes, a finding confirmed in later studies^27-29^. One study on 60 women using Nestorone pellets for up to 24 months found luteal suppression in the first year, preventing ovulation and pregnancy, but ovulation frequency increased in the second year, suggesting temporary ovarian suppression^30^. In Ylänen's study on endometriosis, symptoms recurred post-treatment, with irregular bleeding and hypoestrogenic symptoms being frequently reported^31^.

Evidence on Nestorone pellets is of low quality due to small sample sizes and lack of randomized trials. While studies indicate short-term contraceptive efficacy, adverse effects such as irregular bleeding, hypoestrogenism, and symptom recurrence are common^32,33^. The absence of long-term data limits safety assessment, and current evidence does not support off-label or esthetic use, emphasizing the need for well-designed studies.

### Oxytocin and oxandrolone

No studies involving pellets in humans were found.

## DISCUSSION

Hormonal pellets are increasingly marketed for gynecological issues but are not the first-line treatment. Established alternatives, like gonadotropin-releasing hormone (GnRH) agonists, oral contraceptives, and hormonal intrauterine devices (IUDs), have better safety and efficacy profile for conditions such as endometriosis and uterine fibroids. Evidence for compounded hormonal pellets is mostly of low quality, with outdated studies that do not meet modern standards (Table 3). Concerns about safety include androgenic effects and cardiovascular risks for gestrinone and testosterone, while studies on Nestorone focus on contraception, showing high rates of irregular bleeding. No studies exist for oxytocin and oxandrolone pellets.

**Table 3:** Summary of findings.

Hormone	Grade level	Evidence summary	Limitations
Gestrinone	Very low	Evidence is limited and outdated, primarily from small observational studies with methodological flaws	Small sample sizes, lack of robust data, and frequent adverse effects, including androgenicity and elevated transaminases
Testosterone	Very low	Evidence includes observational studies and a few small randomized trials, showing mixed efficacy in climacteric symptoms and sexual function improvement	Frequent supraphysiologic levels with pellets, androgenic effects, and lack of long-term safety data
Estradiol	Low	Observational studies and small trials suggest benefits for symptom relief and bone density preservation	Adverse effects include endometrial hyperplasia, irregular bleeding, and lack of robust randomized controlled trials (RCTs).
Nestorone	Low	Studies focus on contraceptive use and show short-term ovulation suppression and symptom relief	Frequent irregular bleeding, hypoestrogenic symptoms, and recurrence of symptoms post-treatment; no long-term safety data
Oxytocin	Very low	No clinical studies involving pellets were found	Complete absence of safety or efficacy data in humans
Oxandrolone	Very low	No clinical studies involving pellets were found	Complete absence of safety or efficacy data in humans

The National Academies of Sciences recommends these pellets only when commercial options are unavailable or for patients with allergies^34^. Pellets can cause high hormonal peaks and prolonged elevations, which are not well monitored. Compounded pellets also face challenges in dosage accuracy, release consistency, and sterility. Many supporting studies are outdated, lacking randomization and adequate sample sizes^35^.

In 2021, ANVISA banned gestrinone pellet advertising due to safety concerns, and in 2023, the Federal Council of Medicine regulated their prescription, prohibiting esthetic use. Over 40 medical societies oppose the indiscriminate use of hormonal pellets, reporting over 200 adverse effects, including cardiovascular and psychological issues, as well as irreversible effects in women, like acne and infertility^36-38^.

## CONCLUSION

The available scientific evidence regarding the safety and efficacy of hormones used in subdermal pellets is either of low quality or entirely lacking. Data on their pharmacokinetics remain insufficient, and the well-documented risks associated with conventional hormone therapy cannot be directly extrapolated to these formulations. Prescribing decisions should be guided by high-quality evidence, aligned with established clinical guidelines, and subject to stringent regulatory oversight.

## Data Availability

The datasets generated and/or analyzed during the current study are available from the corresponding author upon reasonable request.
